# Could African and Low- and Middle-Income Countries Contribute Scientifically to Global Cancer Care?

**DOI:** 10.1200/JGO.2015.001032

**Published:** 2015-11-12

**Authors:** Ahmed M. Elzawawy

**Affiliations:** Suez Canal University, Suez Canal region, Ismailia City, Egypt.

Could African countries, as exemplars of low- and middle-income countries (LMICs), make scientific contributions that would increase the affordability of cancer care globally?

After four decades of effort to improve the quality of care, Robert Brook, a distinguished expert on quality, declared in 2010 “The end of the quality improvement movement: long live improving value!”^1^ “Value” is broadly defined as outcomes relative to the total costs of care and encompasses effectiveness, cost-effectiveness, efficiency, quality, safety, and quality of life.^1–3^ Value is relevant to cancer care in both developed and developing countries.^1^ Better value care does not mean inferior care. Economic studies and measures such as cost reduction without regard to the outcomes achieved are dangerous and can lead to false savings and potentially limit effective care.^2,3^

Both the American Society of Clinical Oncology (ASCO) and the European Society for Medical Oncology (ESMO) have been working on ways to define and measure value. In 2014, ASCO's Value in Cancer Care Task Force launched an initiative to define value as the combination of three factors for cancer therapy care: clinical benefit, toxicities, and costs. From the patient's standpoint, value means achieving the best possible outcome consistent with his or her own personal preferences and financial situation.^4^ ASCO recently went a step further and published its ASCO Value Framework to assess value in cancer care and illustrate how value is defined by the patient, health care provider, and payer.^5^ ESMO has unveiled its new tool, the Magnitude of Clinical Benefit Scale, which offers a rational, structured, and consistent approach to stratifying a drug's clinically meaningful benefit. The scale was developed and then field tested in Europe for 77 cancer drugs across 10 cancer types.^6^

The ASCO and ESMO initiatives to define value and clinical benefit should be regarded as important first steps in a long journey to increase the affordability of cancer care in both developed and developing countries. The WHO has called for 80% availability of affordable basic technologies and essential medicines and access to comprehensive health care services by the year 2020.^7^ In fact, 2 years have already passed since the announcement of this plan without any significant progress. Moreover, the superficial policy suggesting that the issue of shortage and affordability of essential cancer drugs will be solved by the use of generics is misleading unless a wider (global) approach, one that mixes innovative and classical approaches, is taken to address the issue.^8,9^

Putting current challenges and barriers aside for the moment, how can African and other LMICs be part of the international scientific solution to providing value instead of being seen as a burden in addressing the affordability of cancer care?^10^

It is well known that within the next 10 years, 70% of patients newly diagnosed with cancer will be living in countries that collectively have only 5% of the global resources for cancer control. It is estimated that, at present, approximately 60% of the world's patients with cancer do not have access to a complete cancer systemic therapy regimen, and the percentage is higher for radiotherapy. The picture is more tragic in Africa,^11,12^ which serves as an example of the wider family of LMICs.

Cancer statistics and registries would be just numbers if those numbers are not viewed as human beings who have (or had) pulsating hearts and hopes. Early detection programs are useless and frustrating to patients, health workers, and authorities if patients cannot afford the kind of basic treatment and adequate, easily accessible supportive and palliative care that will enable them to maintain their (and their family's) social and financial dignity.^12,13^

There is a myth that having national cancer control programs, large national or international meetings, or even national treatment guidelines could be enough to improve the current situation. A WHO survey in 167 countries found that nearly half the countries had some sort of plan for improving treatment, but national guidelines lagged behind so that accessibility and affordability of treatment remained low in developing countries. In fact, national cancer control plans had been designed by copying phrases and text from WHO reports without tailoring each country's plan to its own local conditions and challenges.^14–16^ It is difficult to achieve better value cancer care in the large populations in LMICs without addressing the local realities in a creative way.^10,13,17^ As it stands, the talk about LMICs will continue to contain many slogans and expressions of sympathy, but interventions that truly make a difference will still be limited.

Engaging in innovative strategic thinking and finding new ways to mobilize local resources to improve the availability and accessibility of cancer care are essential to overall and balanced cancer control in underserved countries. African and many other LMICs have at least some local resources, but often they are not used appropriately or are not mobilized. LMICs should not rely entirely on external financial donations from affluent organizations or countries. Instead, what is needed is win-win support and durable assistance from those organizations or countries, as well as pharmaceutical companies. Assistance could take the form of technical support for building local capacity, that is, staff needed for cancer care and research, including cancer care providers, laboratory staff, research coordinators, and data managers. Other types of support might include provision of information and communication technologies, help with obtaining local funds or international grants, instructions on how to collaborate on international work in their own countries, suggestions for ways to provide help and training in managing the financial and secretarial (administrative) aspects of a research project, help with defining ethical considerations in research, and help in editing manuscripts intended for international publications.^13^

What would affluent countries and LMICs have to gain from using a scientific win-win approach? There are important messages on this subject from two influential books. First, “reverse innovation” is pivotal for both high-income countries and LMICs because it implies that affluent countries can make use of innovations coming from LMICs to the benefit of all.^18^ Second, Lord Nigel Crisp described a new vision for global health in the twenty-first century based on our rights and accountabilities as citizens in an interconnected and interdependent world: instead of talking about international development, we should tackle co-development so that rich countries can learn from poorer ones as well as the other way around.^19^ Thus, global oncology is not just about cancer in LMICs, it should be regarded as oncology for the whole world with a special emphasis on cancer care in LMICs because those countries have the majority of the world's population along with the challenges of inadequate cancer care and lack of resources to spend on health.

Conducting more clinical trials in LMICs could shorten the total time needed for conducting clinical trials, may reduce costs, and could enrich the scientific aspects of those trials with more variability. It could also help bring about the sale and use of newer drugs in more cost-effective ways in markets in middle-income and some affluent countries. Such an approach could help companies streamline the development of new drugs and technologies. For the locals, conducting more clinical trials could be a source of income for oncologists, other members of the professional cancer care team, and scientists, and most importantly, it would improve patient care. These elements could all contribute to better value cancer care.^13,17^ There are many opportunities to conduct scientific studies on resource sparing with equal or better outcome, as shown in the following seven examples derived from published studies by many different investigators.^10,13^ (1) Adopting treatment pathways that incorporate evidence-based medicine for patients with non–small-cell lung cancer revealed that evidence-based care resulted in an average cost savings of 35% over 12 months with outcomes equivalent to those of more costly methods of treatment.^20^ (2) There is a need to develop more protocols for intravenous infusion of chemotherapy that require fewer hospitalizations and thus reduce cost. Using less toxic regimens can result in fewer or less severe adverse reactions without compromising the total outcome, thereby reducing the number of unplanned hospital visits (and thus the cost). Using oral administration for chemotherapy could lower transportation costs for patients, eliminate costly drug infusions, reduce the number of hospitalizations, reduce the number of adverse effects resulting from subsequent hospitalizations, and may even improve the quality of life.^10^ New subcutaneous formulations for administering drugs such as trastuzumab (herceptin) could help lower costs for patients.^21^ A new nebulizer device for treating lung cancer with the chemotherapy drug cisplatin could deliver small doses and result in quicker responses without the potential for renal damage that the current intravenous method of administration has.^22^ (3) Pharmacokinetic studies could be performed that focus on lowering drug dose (and therefore the cost) by changing the infusion regimen. The phase I/II trials of prolonged infusion of low-dose gemcitabine are one example. The usual dose of 1,000 to 1,250 mg/m^2^ for one patient might then be enough for 4 to 5 patients with comparable results in responding to solid cancers such as non–small-cell lung cancer and breast, pancreatic, and bladder cancers.^10,23^ (4) Pharmacokinetic studies that focus on drug interaction could be performed. For example, one such study showed that lapatinib for advanced *ERBB2*-positive breast cancer (after treatment with trastuzumab failed) taken orally with food or beverages that contain CYP3A inhibitors (eg, grapefruit juice) and not on an empty stomach as stated on the label resulted in increased plasma levels of lapatinib. This regimen could reduce the dose and eventually reduce the cost of lapatinib by 80%^24^ in addition to saving the burden and cost of treating diarrhea due to unabsorbed lapatinib in the gut when it is taken on an empty stomach.^17^ (5) Interrupted courses of therapy could reduce cost. A phase III randomized trial compared intermittent androgen suppression with continuous androgen suppression in patients with prostate-specific antigen progression after radical radiotherapy. Intermittent androgen suppression was delivered for 8 months in each cycle with restart when prostate-specific antigen reached more than 10 ng/mL off treatment.^25^ (6) Generic equivalents for off-patent drugs could be tested and may result in cost reduction.^8,9^ (7) Scientific studies could be proposed to assess the possibilities of repurposing off-patent drugs and creating new combinations of old drugs. One example is the metronomic use of prolonged low oral doses of cancer drugs.^26^ In a phase II trial, low-dose (6 mg per day) oral estradiol achieved the same response as conventional high-dose (30 mg per day) estradiol in approximately 30% of patients with fewer adverse events in postmenopausal women with aromatase inhibitor–resistant, hormone receptor–positive advanced breast cancer.^27^ The National Cancer Institute (NCI) in the United States created a publically available Web site for at least 5,000 new combinations of 100 approved cancer drugs tested in a cell line panel known as the NCI-60, which is commonly used by cancer researchers worldwide.^28^ This web-available information could become the basis for many future clinical trials of such combinations.^29^ A search of Web sites like that of the Win-Win Scientific Initiative will reveal many other examples of resource-sparing radiotherapy and well-balanced approaches to cancer control that could lead to better value cancer care.^13^

To summarize, affluent countries and international organizations should consider investing in scientific capacity building in LMICs. Institutes in affluent countries could support trained and qualified scientists and others in the health field as paid co-researchers or co-workers who could design studies relevant to the local population and who continue to live and work in their own LMICs. This approach would help prevent the brain drain experienced by LMICs when their most highly qualified people immigrate to the West and it would be one way to ensure that local scientific progress contributes to international knowledge—a win-win situation. This would send a message of cooperation based on scientific evidence and love for humanity and the good side of human beings wherever they are on our planet.^10,13,17,30^ The type of cooperation emphasized throughout this editorial is truly needed when we practice “Global Oncology.”
